# Supplementary material for: SyNDI: synchronous network data integration framework
Source: BMC Bioinformatics. 2018 Nov 6;19:403. doi: 10.1186/s12859-018-2426-5 (PMC6219086; doi:10.1186/s12859-018-2426-5)
Supplement: Supplementary file 5 — Files of the example presented in Additional file 4. This additional file is a zip package that contains all files used in Additional file 4. Detailed descriptions of each of these files are presented in a README file included in this zip package. (GZ 30411 kb) [file 12859_2018_2426_MOESM5_ESM.gz › SyNDITools/Meme2Fimo/meme_4.9.0_4.tar.gz!/meme_4.9.0/doc/meme-format.html]

MEME Suite Motif File Formats


# MEME Suite Motif File Formats

## MEME Output Formats

MEME results are recorded in three file formats:

- HTML and
- plain text,
- XML.

### MEME HTML format

The MEME HTML format is meant to be easy to read and provide useful
visualizations and tools for continuing to work with the discovered
motifs. The HTML output is generated from the XML output by the use
of a XML stylesheet transformation.

### MEME XML format

The MEME XML format is completely specified by the
Document Type Definition (DTD)
found at the start of the MEME XML output. The XML format is intended
for machine reading as it is just the data without any explanatory text
or visualizations. The XML format was added for MEME 4.0.

### MEME plain text format

The MEME plain text format was the original output from MEME and
like the HTML contains explanatory text making it self-documenting.
The minimal MEME format is essentially the MEME text format without
the documentation.

## GLAM2 Output Formats

GLAM2 results are recorded in two file formats:

- HTML and
- plain text.

The GLAM2 format is described in the
Output format section of
the GLAM2 Tutorial.
GLAM2 also provides a MEME minimal motif format
approximation of the motif.

## MEME Suite Motif Input Formats

Most MEME Suite programs, which accept motifs, can use the outputs
of MEME and DREME (plain text, HTML and XML) as well as the
MEME minimal motif format.

The major programs which accept MEME and DREME output are:

- MAST,
- Tomtom,
- GOMO,
- FIMO,
- MCAST,
- SpaMo and
- CentriMo.

GLAM2SCAN will only accept the plain text and HTML forms of GLAM2
output.

## MEME Minimal Motif Format

Users may create motif files in a simplified format for use by the MEME
Suite programs (excluding GLAM2SCAN).

- sample DNA motif and
- sample protein motif.

### Log-odds conversion for MAST now automatic

Prior to release 4.7.0 of the MEME Suite MAST required a log-odds
matrix section to be specified, however the current version
of the MEME Suite is capable of translating the letter-probability matrix
into the log-odds matrix and vice-versa.

### Specification

The Minimal MEME format contains following sections:

1. Version (required)
2. Alphabet (recommended)
3. Strands (optional)
4. Background frequencies (recommended)
5. Motifs (required)

For each motif in the motifs section there are the sub-sections:

1. Motif name (required)
2. Motif letter-probability matrix (recommended\*)
3. Motif log-odds matrix (optional\*)
4. Motif URL (optional)

\*Note that at least one of the two starred sections is required.

#### MEME version line (required)

MEME requires this line to be certain that it really is reading a MEME
motif file and not just something that looks slightly like it. This line
must appear before any other sections in the file.

MEME version version number

The version number should be the MEME
Suite version you are targeting.

For example to target MEME Suite version 4 and above:

MEME version 4

#### Alphabet line (recommended)

The alphabet line tells the MEME Suite what alphabet to expect the
motifs to be in. If this line is not present then the MEME Suite can
attempt to detect this from the background or the motifs themselves.

ALPHABET= alphabet

The alphabet can be ACGT for DNA or
ACDEFGHIKLMNPQRSTVWY for protein.

For example using a DNA alphabet:

ALPHABET= ACGT

#### Strands line (optional)

The strands line only has meaning for DNA motifs and indicates if
motifs were created from sites on both the given and the reverse complement
strands of the DNA sequences. If this line is not supplied then the MEME
Suite will assume that DNA motifs were created from both strands.

strands: which strands

The which strands can be replaced with +
to indicate only the given strand and + - to indicate both strands.

For example to indicate only the given strand was used:

strands: +

#### Background frequencies lines (recommended)

The background frequencies tell the MEME Suite how prevalent each
letter of the motif alphabet was in the source sequences which were used
to create the motifs. If the background frequencies are not supplied then
the MEME Suite will assume uniform background frequencies. The MEME Suite
uses this background to convert between
motif letter-probability matrices and log-odds matrices.

Background letter frequencies (from source):  
letter 1 frequency 1
letter 2 frequency 2
... (repeated) ...
letter n-1 frequency n-1
letter n frequency n

The source is not required and if you
wish you can leave off then end of the first line after "Background
letter frequencies". On the next line is listed each letter in the
alphabet followed by its frequency. The letters must be listed in the same
order as in the alphabet line and the frequencies should sum to 1.

An example of uniform DNA frequencies:

Background letter frequencies  
A 0.25 C 0.25 G 0.25 T 0.25

An example of protein frequencies with a source listed:

Background letter frequencies (from lipocalin.s):  
A 0.071 C 0.029 D 0.069 E 0.077 F 0.043 G 0.057 H 0.026 I 0.048 K 0.085   
L 0.087 M 0.018 N 0.053 P 0.032 Q 0.029 R 0.031 S 0.058 T 0.048 V 0.069   
W 0.017 Y 0.050

#### Motif name line (required)

The motif name line indicates the start of a new motif and designates
an identifier for it which much be unique to the file. It also allows for
an alternate name which does not have to be unique.

MOTIF identifier alternate name

For example:

MOTIF MA0002.1 RUNX1

#### Motif letter-probability matrix lines (recommended)

The letter probability matrix is a table of probabilities where the
rows are positions in the motif and the columns are letters in the
alphabet. The columns are ordered alphabetically so for DNA the first
column is A, the second is C, the third is G and the last is T. For
protein motifs the columns come in the order A, C, D, E, F, G, H, I, K,
L, M, N, P, Q, R, S, T, V, W and Y. As each row contains the probability
of each letter in the alphabet the probabilities in the row must sum to 1.
**If this section is not specified then the log-odds matrix must be
specified**.

letter-probability matrix:
alength= alphabet length
w= motif length
nsites= source sites
E= source *E*-value  
... (letter-probability matrix goes here) ...

All the "key= value" pairs after the "letter-probability
matrix:" text are optional. The "alength=
alphabet length" and
"w= motif length" can be derived
from the matrix if they are not specified, provided there is an empty line
following the letter probability matrix. The "nsites=
source sites" will default to 20 if it
is not provided and the "E=
source *E*-value" will default to
zero. The source sites is used to apply
pseudocounts to the motif and the
source *E*-value is used for filtering
the motifs input to some MEME Suite programs (see MAST's -mev option).

An example of a DNA motif's letter-probability matrix:

letter-probability matrix: alength= 4 w= 18 nsites= 18 E= 1.1e-006  
0.611111 0.000000 0.055556 0.333333  
0.555556 0.000000 0.111111 0.333333  
0.222222 0.166667 0.222222 0.388889  
0.000000 0.111111 0.000000 0.888889  
0.000000 0.055556 0.944444 0.000000  
0.111111 0.000000 0.000000 0.888889  
0.055556 0.000000 0.888889 0.055556  
0.833333 0.111111 0.055556 0.000000  
0.111111 0.388889 0.277778 0.222222  
0.333333 0.055556 0.500000 0.111111  
0.111111 0.222222 0.111111 0.555556  
0.277778 0.222222 0.222222 0.277778  
0.111111 0.055556 0.722222 0.111111  
0.388889 0.166667 0.055556 0.388889  
0.055556 0.000000 0.111111 0.833333  
0.055556 0.777778 0.000000 0.166667  
0.777778 0.000000 0.222222 0.000000  
0.277778 0.611111 0.055556 0.055556

#### Motif log-odds matrix lines (optional)

If you've included the letter-probability matrix then unless you want
MAST to use a specific log-odds matrix then **there is no reason to
include this section**. The original version of MEME only output motifs
with a log-odds matrix. Later on the letter-probability matrix was added
and MEME and MAST were merged with Meta-MEME to become the MEME Suite.
Since then all new programs have been written to use the
letter-probability matrix leaving MAST as the only program which uses it.
In the output from MEME the log-odds matrix has some additional tweaks
(especially in protein motifs) which are impossible to perform with just
the letter-probability matrix and the 0-order background so it has been
kept around.

The log-odds matrix is a table of scores where the rows are positions
in the motif and the columns are letters in the alphabet.

The scores are calculated as follows:

 `let bL be the background probability for letter L  
let pLi be the probability for the letter L at position i in the motif  
let sLi be the score for the letter L at position i in the motif  
sLi = round((log(pLi / bL) / log(2)) * 100)` 

As the log of 0 is negative infinity then pseudocounts should be added
to the probabilities first.

log-odds matrix:
alength= alphabet length
w= motif length
E= source *E*-value  
... (log-odds matrix goes here) ...

As in the letter-probability matrix the "key= value" sections
are optional and have the same defaults

An example of a DNA motif's log-odds matrix:

log-odds matrix: alength= 4 w= 18 E= 1.1e-006  
  101  -1081   -182     13  
   87  -1081    -82     13  
  -45    -23     18     35  
-1081    -82  -1081    155  
-1081   -182    227  -1081  
 -145  -1081  -1081    155  
 -245  -1081    218   -245  
  145    -82   -182  -1081  
 -145     99     50    -45  
   13   -182    135   -145  
 -145     18    -82     87  
  -13     18     18    -13  
 -145   -182    188   -145  
   35    -23   -182     35  
 -245  -1081    -82    145  
 -245    199  -1081    -87  
  135  -1081     18  -1081  
  -13    164   -182   -245

#### Motif URL line (optional)

The URL line specifies a web-page to link to when mentioning the motif
in results.

URL web page URL

For example:

URL http://jaspar.genereg.net/cgi-bin/jaspar\_db.pl?ID=MA0002.1&rm=present&collection=CORE
